# Society Meetings

**Published:** 1902-11

**Authors:** 


### SOCIETY MEETINGS.

### Buffalo Academy of Medicine.

PROGRAM, 1902-1903.

September 2, Surgery—Surgical treatment of tic douloureux, W. C. Phelps. Presentation of cases, Marcell Hartwig. Discussion, W. C. Krauss.

September 9, Medicine—Stated meeting of the Academy. Syphilis of the nervous system, F. S. Crego. Cerebral hemorrhage following injury to the spine, Sydney A. Dunham.

September 16, Pathology—On the nature and germicidal action of the organic peroxides, F. G. Novy, Ann Arbor.

September 23, Obstetrics and Gynecology—Movable kidney; frequency, method of diagnosis and treatment, A. L. Benedict. Report of cases. (1) Tetanus following delivery; (2) Rupture of the uterus, Irving W. Potter.

September 30, Ophthalmology, etc.—Adenoids, Carl Leo-Wolf, Niagara Falls. Some of the more common forms of ocular syphilis, Arthur G. Bennett. Presentation of cases of sinus thrombosis, Geo. A. Sloan. Radical antral operation, and partial extirpation of the larynx. Exhibition of patients, Geo. F. Cott.

October 7, Surgery—Subject to be announced, Eugene A. Smith.

October 14, Medicine—The works of Edward Jenner, and their value in the modern study of smallpox, George A. Dock, Ann Arbor.

October 21, Pathology—Subject to be announced, George Blumer, Albany. A case of unusual injury of the head, recovery, subsequent autopsy, Henry P. Frost. Trichinosis, Edwin R. Gould.

October 28, Obstetrics and Gynecology—Prophylaxis and treatment of puerperal infection, John V. Woodruff. Infant feeding, A. J. Colton.

November 4, Surgery—Stated meeting of the Academy. Subject to be announced, Roswell Park.

November 11, Medicine—Subject to be announced, George M. Gould, Philadelphia.

November 18, Pathology—On the relation of human and bovine tuberculosis, Veranus A. Moore, Ithaca.

November 25, Obstetrics and Gynecology—Pelvic abscess and its treatment, Herman E. Hayd. A nursing mother, G. W. Taylor. Period of dentition, H. B. Singer.

December 2, Surgery—Subject to be announced, John Parmenter.

December 9, Medicine—Physical diagnosis of cardiac defects, Joseph Sailer, Philadelphia. Stimulation, Eli H. Long. Nephritis in infants, C. S. Jones.

December 16, Pathology—The rôle of the mosquito in yellow fever, Major Walter Reed, Washington.

December 23, Obstetrics and Gynecology—Indications for inducing abortion, Marcell Hartwig. Hodgkin's disease in children, Dewitt Sherman.

December 30, Ophthalmology, etc.—Subject to be announced, H. V. Wurdemann, Milwaukee. Inoculation of smallpox in the rabbit eye, Harvey R. Gaylord.

January 6, Surgery—Subject and essayist to be announced.

January 13, Medicine—Floating kidney, Charles Cary. Subject to be announced, Irving M. Snow.

January 20, Pathology—Stated meeting of the Academy. Intestinal paresis in man, Charles W. Filler.

January 27, Obstetrics and Gynecology—Diagnosis of exceptional conditions in labor, Stephen Y. Howell. Report of anomalous cases, Henry G. Bentz. Ancient gynecology, Henry D. Ingraham.

February 3, Surgery—Subject to be announced, George M. Edebohls.

February 10, Medicine—Infantile eclampsia, A. J. Cotton. Exophthalmic goitre, G. Himmelsbach.

February 17, Pathology—Malformation of female pelvis, J. Whitridge Williams, Baltimore.

February 24, Obstetrics and Gynecology—Deformities of the female pelvis, P. W. Van Peyma. Subject to be announced, C. C. Frederick.

March 3, Surgery—Subject to be announced, Benjamin Graves, Boston.

March 10, Medicine—Cardiac disease, H. C. Buswell. Subject to be announced, A. L. Benedict.

March 17, Pathology—Valvular lesions of the heart, Wm. S. Thayer, Baltimore.

March 24, Obstetrics and Gynecology—Why minor gynecological operations fail to give anticipated results, S. Goldberg. Subject to be announced, Earl P. Lothrop.

March 31, Ophthalmology, etc.—Stated meeting of the Academy. The reduction of the eye in cave animals, Carl H. Eigenmann, Bloomington, Ind.

April 7, Surgery—Subject and essayist to be announced.

April 14, Medicine—Report of thirty cases of pernicious anemia, Charles G. Stockton, and A. E. Woehnert. Subject to be announced, Julius Ullman.

April 21, Pathology—Subject to be announced, A. E. Woehnert.

April 28, Obstetrics and Gynecology—Injuries to the parturient canal during labor; their prevention and treatment, C. E. Congdon. Subject to be announced, L. G. Hanley.

May 5, Surgery—Subject and essayist to be announced.

May 12, Medicine—Splenic anemia, Irving P. Lyon. Subject to be announced, Delancey Rochester.

May 19, Pathology—Biological test for the blood of different species, Irving P. Lyon.

May 26, Obstetrics and Gynecology—Stated meeting of the Academy. Relative indications for Cesarean section, symphysiotomy and perforation, M. D. Mann.

June 2, Ophthalmology, etc.—Adenoids from the standpoint of the general practitioner, Carl G. Leo-Wolf, Niagara Falls. Pharyngitis, Frank W. Hinkel.

June 9, annual meeting.

---

THE Southern Surgical and Gynecological Association will hold its twelfth annual meeting at Cincinnati, November 11-13, 1902, under the presidency of Dr. William E. B. Davis, of Birmingham. The preliminary program has been issued announcing 36 papers, which deal with a great variety of subjects full of interest to both surgeons and gynecologists. A large attendance is anticipated, this being the first meeting of the association north of the Ohio river. The railroads grant the concession of a fare and a third. It is expected the headquarters of the association will be at the Grand Hotel, in which also the meetings will be held.

Dr. Thaddeus A. Reamy, the veteran gynecologist, is chairman of the committee of arrangements, and Dr. William D. Haggard, of Nashville, is the secretary. Either of these officers may be addressed in regard to details of the meeting—the first-named as to accommodations and the like, and the second-named in regard to the program.

---

The New York State Medical Association held its annual meeting at New York, October 20-23, 1902, under the presidency of Dr. A. A. Hubbell, of Buffalo. The officers elected for the ensuing year were: president, Frederick H. Wiggin, New York; vice-president, William H. Thornton, Buffalo; secretary, Guy D. Lombard, New York; treasurer, E. H. Squibb, New York.

---

The Vermont Society for the Study and Prevention of Tuberculosis was organised at Burlington, September 28, 1902. Officers were elected as follows: president, Hon. E. C. Smith, of St. Albans; vice-presidents, Drs. Don D. Grout, of Waterbury, and C. W. Peck, of Brandon; secretary, Dr. H. E. Lewis, of Burlington; treasurer, H. L. Stilson, of Bennington; executive committee, Drs. H. D. Holton, of Brattleboro, W. M. Platt, of Shoreham, Henry Ballard, of Burlington, E. H. Ross, of St. Johnsbury.

---

The Medical Association of Central New York held its 35th annual meeting at Syracuse, October 21, 1902, under the presidency of Dr. A. L. Beahan, of Canandaigua. The program contained a list of 18 papers, most of which were read either in detail or by title, and will be printed in this Journal.

---

The Oregon State Medical Society held its annual meeting September 11, 1902, when the following-named were elected officers: president, Henry Waldo Coe, Portland; first vice-president, F. W. Van Dyke, Grants Pass; second vice-president, J. A. Geisendorfer, The Dalles; third vice-president, J. P. Tamiesie, Hillsboro; secretary, A. D. Mackenzie, Portland; treasurer, Mae H. Cardwell, Portland.

